# Wearable Immersive Virtual Reality Device for Promoting Physical Activity in Parkinson’s Disease Patients

**DOI:** 10.3390/s22093302

**Published:** 2022-04-26

**Authors:** Pablo Campo-Prieto, José Mª Cancela-Carral, Gustavo Rodríguez-Fuentes

**Affiliations:** 1 Department of Functional Biology and Health Sciences, Faculty of Physiotherapy, University of Vigo, 36005 Pontevedra, Spain; pcampo@uvigo.es (P.C.-P.); gfuentes@uvigo.es (G.R.-F.); 2 HealthyFit Research Group, Galicia Sur Health Research Institute (IIS Galicia Sur), SERGAS-UVIGO, 36213 Vigo, Spain; 3 Department of Special Didactics, Faculty of Education and Sports Science, University of Vigo, 36005 Pontevedra, Spain

**Keywords:** wearable technology, physical activity, Parkinson´s disease, movement/mobility, physiotherapy, neurological disorders, virtual reality exposure therapy, upper limb function, rehabilitation, disease management

## Abstract

Parkinson’s disease (PD) is a neurological disorder that usually appears in the 6th decade of life and affects up to 2% of older people (65 years and older). Its therapeutic management is complex and includes not only pharmacological therapies but also physiotherapy. Exercise therapies have shown good results in disease management in terms of rehabilitation and/or maintenance of physical and functional capacities, which is important in PD. Virtual reality (VR) could promote physical activity in this population. We explore whether a commercial wearable head-mounted display (HMD) and the selected VR exergame could be suitable for people with mild–moderate PD. In all, 32 patients (78.1% men; 71.50 ± 11.80 years) were a part of the study. Outcomes were evaluated using the Simulator Sickness Questionnaire (SSQ), the System Usability Scale (SUS), the Game Experience Questionnaire (GEQ post-game module), an ad hoc satisfaction questionnaire, and perceived effort. A total of 60 sessions were completed safely (without adverse effects (no SSQ symptoms) and with low scores in the negative experiences of the GEQ (0.01–0.09/4)), satisfaction opinions were positive (88% considered the training “good” or “very good”), and the average usability of the wearable HMD was good (75.16/100). Our outcomes support the feasibility of a boxing exergame combined with a wearable commercial HMD as a suitable physical activity for PD and its applicability in different environments due to its safety, usability, low cost, and small size. Future research is needed focusing on postural instability, because it seems to be a symptom that could have an impact on the success of exergaming programs aimed at PD.

## 1. Introduction

Parkinson’s disease (PD) is a neurological disorder that usually appears in the 6th decade of life and affects up to 2% of older people (65 years and older). Its global prevalence is expected to double by 2040 [1]. Its therapeutic management is complex and includes not only pharmacological therapies but also other types of therapies, such as physical activity and multidisciplinary rehabilitative treatment [2,3,4,5].

Exercise therapies have shown good results in PD, as rehabilitation and maintenance of physical and functional capacities are crucial aspects in these patients [6]. In PD management, physiotherapy is mainly an exercise-based intervention that addresses five core areas: physical fitness, transfers, manual activities, balance, and gait [7]. Rehabilitation activities that are more engaging, e.g., virtual reality (VR), can be more effective compared to conventional rehabilitation [8], since they can enhance adherence and long-term use. This is important if we talk about exercise therapies, where the benefits achieved quickly disappear if the therapies are not undertaken regularly [9].

Exergames require the active participation of the user in performing movements and physical tasks that can reproduce the results of traditional rehabilitation strategies in PD [10]. Systematic reviews and meta-analysis have shown their potential to improve balance and quality of life and reach high levels of satisfaction and adherence in PD populations [11,12,13]. Gaming augmented with visual and audio feedback exploits neurophysiological reward mechanisms e.g., by engaging dopaminergic reward systems, which can enhance brain plasticity [14,15].

Immersive virtual reality (IVR) refers to technologies that enhance this positive feedback by providing a first-person perspective system through a head-mounted display (HMD), which allows users to experience virtual worlds in a realistic fashion. An HMD represents one of the most immersive VR technologies and relies on multisensory approaches that foster immersion into the experience or game [16]. Although applications of exergame-based exercise programs with HMDs targeting PD remain relatively unexplored, they have often been carried out with systems -HTC Vive Pro- (HTC Corporation, Taoyuan City, Taiwan) that require significant and expensive additional equipment, mainly a high-performance computer, a monitor, and a base station system to set up a definitive game space, and furthermore have been tested on small samples and in studies consisting of only a few sessions. In addition, the studies of exergames applied to the Parkinson’s population have focused on those that require to perform motor tasks, mainly involving lower and upper limb mobility, which is often affected in these patients [9].

However, today, there are inexpensive options on the market (EUR 349–449) in wearable formats that do not need the support of an additional gaming computer. Facebook’s Oculus Quest 2 could be one of these options.

FIT-XR is a commercial exergame that could be suitable for people with PD as it offers various training sessions focused on physical activity and general body mobility. It also has characteristics comparable with those of traditional physical rehabilitation treatments of PD-focused physiotherapy, for example, joint mobility, balance, and multicomponent exercise (combination of endurance, resistance, or flexibility exercise with balance, gait, agility, or proprioceptive training) [10].

The overall aim of this study is to explore whether this commercial wearable HMD hardware and the IVR exergame proposed could be feasible for people with PD. We report on its safety, usability, personal experiences, user satisfaction, and effort post exergame.

## 2. Materials and Methods

### 2.1. Participants

A heterogeneous sample of 32 volunteers (78.1% men; 71.50 ± 11.80 years) diagnosed with PD (2 ± 1 Hoehn and Yahr stage; 5.97 ± 5.53 time since diagnosis) and belonging to the Vigo Association of Parkinson (Vigo, Spain) were part of the study. Taking into account previous research [17], the following exclusion criteria were established: inability to correctly respond to the assessment protocol according to the clinician´s judgment; the presence of cardiovascular, pulmonary, or musculoskeletal conditions that according to the physiotherapist´s judgment would affect the patient’s ability to participate in the study; and the presence of severe visual loss that could interfere with the ability to see the IVR exergame as well as possibly provoke vertigo, epilepsy, or psychosis. Recruitment was carried out by the association’s physiotherapist.

The study procedure was approved by the Ethics Committee of the Faculty of Physiotherapy of the University of Vigo Institutional Review Board (no. 205-2021-8), and all participants provided signed informed consent at the beginning of the study.

### 2.2. Wearable Device

**Figure 1:**
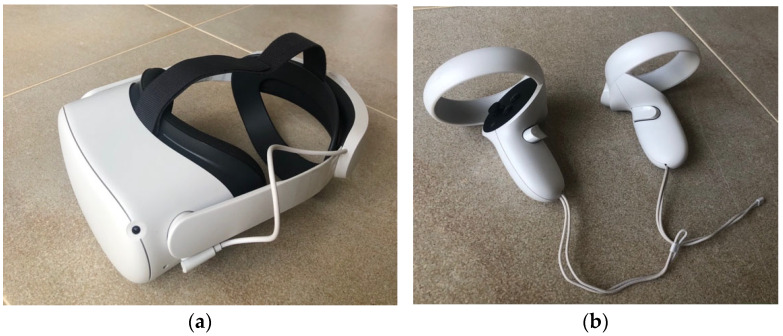
Wearable device used in the study. (**a**) HMD Oculus Quest 2 + Elite strap and (**b**) controllers.

The equipment selected was the HMD Oculus Quest 2 (Oculus VR, Menlo Park, CA, USA). This is a standalone system needing only 2 controllers and a WIFI network for its operation. It is an option more portable and economical than the classic models (Rift from Oculus or Vive from HTC) as it does not need a supporting gaming PC or base stations to set up the gaming area. We decided to add additional equipment (an elite strap), which, in addition to being more ergonomic, incorporates an additional battery to ensure greater autonomy of play, and an Apple iPad 10” so that the physiotherapist could follow the progress of the task via the Oculus App with greater ease. The iPad allowed the therapist to see what the patient sees by mirroring the headset screen. Figure 1 shows the VR device used.

A play area of approximately 5 m^2^ was marked out following the manufacturer’s recommendations and based on our previous experience [17].

### 2.3. The Exergame

Screening was first undertaken by the author (P.C.), who has extensive gaming experience, by systematically browsing the Oculus store. VR games were ordered by popularity and content (fitness, kinetic sports, competition, and music games). Of those deemed suitable, P.C. searched for game clips and reviews and, if available, played the demos. Games that were promising enough were acquired, downloaded, and tested. P.C. is a clinical physiotherapist and a specialist in PD management. Due to the cumulative experience in our previous work employing similar exergames [17,18], FIT-XR was deemed suitable.

**Figure 2:**
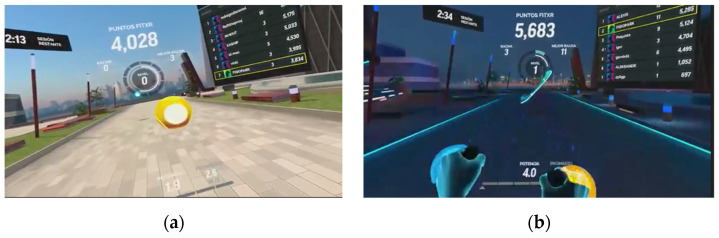
Screenshots of different scenarios of FIT-XR. (**a**) Balls to train upper limbs; (**b**) obstacles to train lower limbs.

In previous studies, we have shown that virtual scenarios without accelerations or sudden changes of view minimize the impact of cybersickness. FIT-XR is available in the Oculus store (https://www.oculus.com, accessed on 15 January 2022). It is a game that re-creates a virtual gym and offers different interfaces (indoor or outdoor scenarios). It offers 3 workout modes: boxing, high-intensity interval training (HIIT), and dance. The exergame was thoroughly tested and scrutinized in our lab. We checked all settings and play modes. HIIT modes were ruled out as a precaution because they were too demanding in terms of effort and the dance mode because it required coordination tasks of high difficulty. After being tested by the other authors and by 2 physiotherapists with PD experience, the boxing mode was considered a suitable exergame option for the target group in this study (Figure 2).

According to research that places boxing as a promising physical therapy in PD [19,20], with possible benefits in terms of balance, mobility, and quality of life and also in gait, speed, and endurance, we finally chose the boxing mode in FIT-XR. The participant has to hit the balls that appear; in the direction that is indicated; and the faster the player hits the balls, the higher the score. The hand controllers represent boxing gloves in this immersive game. In addition, the player must dodge elements that provoke lateral movements of the trunk, weight transfers, and squats. The gameplay itself does not require the user to press any buttons, and although it was designed to be played while standing, it can also be played in a sitting position.

The physical therapist gave a pre-test demonstration to each participant and explained how to perform the movements (intensity and range of motion) to avoid possible injuries. In addition, all sessions were guided and supervised by the therapist, who gave feedback all the time.

### 2.4. Intervention

A game zone was set aside in the gym of the association, where 60 boxing game sessions were carried out using the option “The Clock” (recommended for beginners and lasting 3 min). This option was considered suitable under the clinical criteria of the physiotherapist, since physical tasks proposed must be according to the capacities of the PD patients. The training must be initially affordable (duration, intensity, and frequency) and allow attainable objectives so the participant is not demotivated [10]. In addition, this option is static (it keeps the feet in the same position all the time), thus preventing injuries from occurring when participants forget about the real environment and the tasks can be performed safely in small spaces.

**Figure 3:**
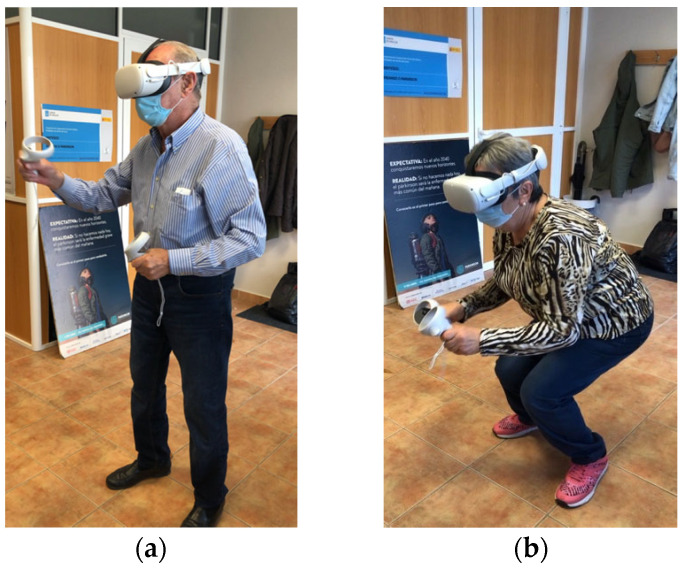
Participants during individual sessions. (**a**) Patient train for speed and strength in upper limbs while hitting balls. (**b**) Participant train for balance and endurance in lower limbs while avoiding obstacles.

There was an initial pre-session time for warm-up (joint mobility) and a final post-session time (stretching). In total, 28/32 participants performed two training sessions, with a rest interval in between (Figure 3). All sessions were guided and supervised by the center’s physiotherapist.

### 2.5. Assessments

Considering the research objectives, after each session, the safety of the intervention was evaluated using the Simulator Sickness Questionnaire (SSQ, adapted and translated into Spanish) [21], the usability of the system with the System Usability Scale (SUS) [22], and personal experiences with the Game Experience Questionnaire (GEQ-post game module) [23] and an ad hoc satisfaction questionnaire. Participants scored their perceived physical exertion after every training session using the Borg Rating of Perceived Exertion [24], and the total score of the game was recorded. All these tools have been used in previous research in this field [25,26].

### 2.6. Statistical Analysis

The data were analyzed using the Statistical Package for the Social Sciences (SPSS Inc., Chicago, IL, USA) for MAC version 25.0. Descriptive statistics were generated for all variables, and distributions of variables were expressed as the mean ± the standard deviation and percentage. The Kolmogorov–Smirnov tests were used to assess the normality of distribution for each variable. Variables were normally distributed (Kolmogorov–Smirnov tests, all *p* < 0.05). A one-factor ANOVA test with post hoc analyses (Bonferroni) was applied to identify the influence of the stage of the disease (I, II, or III) on the VRI task performed, and a one-factor ANOVA test with post hoc analyses (Bonferroni) was applied to identify the influence of the first symptom diagnosed (tremor, bradykinesia-rigidity, postural instability, etc.) on the VRI task performed. Statistical significance was set at *p* < 0.05.

## 3. Results

The demographic and clinical characteristics of the patients are shown in Table 1. All participants completed at least one session, and 28/32 completed two. No adverse effects were observed during or after the training sessions. Table 2 shows the results of safety, usability, and personal experiences.

Regarding levels of user satisfaction, participants were overwhelmingly positive when asked about their thoughts on the training sessions. The ad hoc questionnaire includes the following questions: How was the experience? What did you like the best? Was there anything you didn´t like? Would you repeat the experience with IVR? Would you recommend the experience of IVR? Do you think the IVR exercise is suitable for people with PD? Why? and Free comments.

In all, 88% of the sample expressed that the training was “good” or “very good.” The participants also reported that the exergame was a useful tool for their disease (100%) and they would recommend it for people with PD (100%). They often mentioned their improvement in the second session. Table 3 shows the exergame score and the perceived effort for each session, and Table 4 and Table 5 show the relationship between the Hoehn and Yahr stage and the first symptom diagnosed with task performance, respectively.

## 4. Discussion

Our outcomes are exciting, showing that it is possible to carry out boxing exergame sessions combined with a wearable commercial HMD for use in PD.

Until now, our investigations have been with VR devices that needed additional equipment (HTC Vive Pro), making it difficult to relocate the trials. The system used in this study has allowed us to move our trial to any place comfortably, in this case, to the Vigo Association of Parkinson, with VR equipment that fits in a small bag.

Furthermore, no adverse effects were reported (no cybersickness symptoms). This fact is important and is consistent with previous research [17,18] where games were used with similar aims although they were tested over only a few sessions, whereas in our study, there was a total of 60 sessions. In addition, the stability of the wearable device allowed the safe completion of the game by all participants, without any incidents or injuries.

Even so, in the current study, the session times carried out were relatively short and it would be necessary to verify that the safety results demonstrated here are maintained in longer immersive sessions.

Equipment usability was also satisfactory (SUS >75%). These results are in line with previous findings [27]. Anyway, in our opinion, the Oculus Quest 2 hand controllers may have made the virtual tasks easier for these patients, who often have symptoms affecting the upper limb in general and eye–hand in particular [28], as they are objectively smaller and lighter and, therefore, more manageable than the HTC Vive Pro hand controllers. In turn, carrying an object such as a controller could ensure better tremor self-management, although it would be interesting if future research involves games with hand-tracking setups.

The dimensions of the GEQ-post game reinforced the proposal. Although the results for positive experiences were only moderately satisfactory (2.18/4), the metrics determining aspects such as negative experiences (0.01/4), fatigue (0.09/4), or return to reality (0.03/4) reinforce the suitability and safety of the chosen exergame.

In addition, the fact that the satisfaction opinions emphasize the usefulness of the game for the treatment of PD, and also the subjects´ willingness to use it frequently, could suggest a stronger adherence to future treatments with these virtual tools. The usability of the selected wearable device and the positive opinions generated interest in many of the participants around the possibility of acquiring a device and using it at home.

Similarly, as expected, in the second session, individual performance improved, with higher scores and perceived level of effort in 100% of the sample. The participants in the advanced stage of the disease also improved their performance. As expected, the stage of the disease influenced performance, especially in the score achieved in both sessions (*p* < 0.05). This fact seems reasonable since stage III patients present more limitations in their physical capacities, mainly in postural instability and balance [29].

Although we consider that this fact is not too important, we do, however, think that what really is relevant is the fact that all of them successfully completed the sessions, improving their scores and their levels of effort regardless of the stage and evolution of the disease.

Although it was not our objective, an interesting finding was the relationship between the first symptom of PD and the results of the exergame. In our case, individuals whose first symptom of PD was postural instability performed less well than those who started with tremor or bradykinetic-rigid signs and significantly less well (*p* < 0.05) than those who presented other symptoms in the first diagnosis. These findings add to what was previously commented on the stage of the disease, seeming to indicate that postural instability plays an important role in the proposed virtual tasks. Therefore, they open the door to future research with a view to exploring how the first symptom diagnosed or balance disturbances influence the performance of traditional and/or virtual physical tasks.

Moreover, as in other studies with IVR and PD [30,31], the work carried out in FIT-XR has been with boxing, which is fundamentally based on joint mobility, muscle power, muscle tone, perception, balance, and energy and also on the ability to respond quickly to unexpected stimuli. This is relevant since it works on cognitive domains such as decision making, which are often deficient in PD due to the impairment of executive functions.

Some recent studies with IVR have found physical and cognitive benefits in elderly people [32]. So testing this aspect in people with PD represents a challenge for future research.

In any case, future research with higher methodological quality and with longer interventions would be necessary to corroborate the safety of our proposal. It would also be advisable to evaluate the aspects to be improved in PD, such as balance, bradykinesia, and tremor.

### Limitations

These outcomes are promising, but there are some limitations. The first limitation is that, although the sample may be relevant in terms of number, it may not be representative of the population of patients with Parkinson’s disease, which could lead to recruitment bias. A second limitation is that there were only two sessions per person, so we cannot determine adherence and safety to IVR exposures in longer interventions. A third limitation is that specific physical-functional capacities were not evaluated, so we cannot point out exactly what the improvement in the game score seen in our study is beyond the motivating effect it has had on the patients as a whole. Therefore, future research should be designed on the basis of clinical trials of several weeks’ duration, with two or three sessions per week and an evaluation of objective physical-functional capacities, in addition to carrying out a follow-up that would allow us to analyze the time it takes for the possible therapeutic benefits to be achieved.

## 5. Conclusions

The results of our study support the use of the FIT-XR exergame in the PD population and position the Oculus Quest 2 HMD as a suitable wearable device for physical activity and with which to carry out future rehabilitation programs.

First, this is because it was feasible to carry out training with PD patients, regardless of the evolutionary stage of their disease, showing a good level of safety (no adverse effects) in the 60 training sessions performed. Although it was not our research objective, it seems important to pay attention to postural instability. This is a key symptom in the progression of the disease and could influence the selection criteria of the sample in future studies, e.g., maybe the standing or sitting position of the user. In any case, as we have already highlighted, the most relevant from the clinical point of view is that all the patients completed the task without adverse effects.

Second, this is because of the ease of using the equipment correctly, its easy installation almost anywhere, and the positive opinions of the participants.

Demonstrating the feasibility of exercise programs—designed for patients with neurological conditions—to be undertaken whilst wearing inexpensive wearable technological devices could be advantageous for associations of patients with Parkinson’s, Alzheimer’s, or other related disorders, which often have limited resources. In the future, these devices could become useful supporting tools, with many potential benefits, for patients´ physical, psychological, and social rehabilitation and, due to their portable design, could allow itinerant therapies at home itself.

## Figures and Tables

**Table 1:** Demographic and clinical characteristics of the patients (n = 32).

		Mean	SD	%
**Age** (years)		71.50	11.80	
**Gender**	Female			21.9%
	Male			78.1%
**Height** (m)		1.67	0.08	
**Weight** (kg)		75.88	13.37	
**BMI** (m/kg^2^)		27.20	5.08	
**H&Y stage**		2	1	
**Time since diagnosis** (years)		5.97	5.53	
**First symptom**	Tremor			40.6%
	Bradykinesia-rigidity			43.8%
	Postural instability			6.3%
	Other			9.4%
**Motor fluctuation**				46.9%
**Dyskinesias**				9.4%
**Freezing**				37.5%

BMI: body mass index; H&Y: Hoehn and Yahr; SD: standard deviation.

**Table 2:** Results of safety, usability, and post-game experiences.

	Mean	SD
**SSQ_ST**	0.00	0.00
**SUS_ST**	75.16/100	7.46
**GEQ-post game** **(Positives experiences)**	2.18/4	0.66
**GEQ-post game** **(Negative experiences)**	0.01/4	0.03
**GEQ-post game** **(Tiredness)**	0.09/4	0.30
**GEQ-post game** **(Return to reality)**	0.03/4	0.13

GEQ: Game Experience Questionnaire; SD: standard deviation; SSQ: Simulator Sickness Questionnaire; SUS: System Usability Scale.

**Table 3:** Total scores (FIT-XR and perceived effort) for each session.

	Mean	SD
**Total FIT-XR score** **(Session 1)**	6973	10,887
**Borg score** **(Session 1)**	4	2
**Total FIT-XR score** **(Session 2)**	14,797	17,999
**Borg score** **(Session 2)**	5	2

SD: standard deviation.

**Table 4:** Relationship between disease stage and VRI task performance.

Stage	I		II		III		ANOVA
	Mean	SD	Mean	SD	Mean	SD	
**FIT-XR score** **(Session 1)**	13,189 ^$^	13,733	3756	5822	729	1124	F = 5.266; * *p* = 0.011
**Borg score** **(Session 1)**	4	2	3	2	4	2	F = 0.376; *p* = 0.690
**FIT-XR score** **(Session 2)**	23,876 ^$^	20,550	9945	13,202	2640	3271	F = 4.701; * *p* = 0.019
**Borg score** **(Session 2)**	5	2	6	3	5	2	F = 0.413; *p* = 0.666

^$^: Significant differences between stages I and III. * Statistical significance. SD: standard deviation.

**Table 5:** Relationship between the first symptom diagnosed and VRI task performance.

	First Symptom Diagnosed								
	Tremor		Bradykinesia Rigidity		Postural Instability		Other		ANOVA
	Mean	SD	Mean	SD	Mean	SD	Mean	SD	
**FIT-XR score** **(Session 1)**	5309	8433	9707	14,060	221 ^#^	156	5920	2787	F = 2.939; * *p* = 0.040
**Borg score** **(Session 1)**	3	2	5	2	5	1	2	1	F = 0.659; *p* = 0.585
**FIT-XR score** **(Session 2)**	14,130	15,898	18,546	21,861	269 ^#^	117	10,459	6004	F = 3.012; * *p* = 0.043
**Borg score** **(Session 2)**	5	2	5	2	6	3	3	1	F = 1.093; *p* = 0.371

^#^: Significant differences between postural instability and other symptoms. * Statistical significance. SD: standard deviation.

## Data Availability

The data presented in this study are available on request from the corresponding author.
